# Dystrophic Calcification in the Oral Cavity Resulting in Mechanical Dysphagia: A Case Report and Review of Calcification in the Head and Neck Region

**DOI:** 10.7759/cureus.7469

**Published:** 2020-03-30

**Authors:** Anup Kumar G, Shakti Singh Deora

**Affiliations:** 1 Oral and Maxillofacial Surgery, Noorul Islam College of Dental Science, Trivandrum, IND; 2 Oral and Maxillofacial Surgery, OOM Superspeciality Head & Neck Surgical Centre, Ahmedabad, IND

**Keywords:** dystrophic, calcification, idiopathic, dysphagia, sialolith, tonsilolith, carotid calcification

## Abstract

Soft tissue calcifications in the oral cavity and maxillofacial region are most often detected as incidental findings on routine radiographic examination. But sometimes these soft tissue calcifications can be serious and may need treatment or follow-up of the underlying cause. Deposition of calcium salt as a result of chronic inflammation, necrosis or scarring in injured tissues despite normal phosphorous and calcium metabolism is called dystrophic calcification. A variety of systemic disorders can be associated with this type of calcification but, still, the pathophysiology is not clear. Here we present a case of dystrophic calcification in the floor of the mouth of an 18-year-old female patient associated with dysphagia which was excised by intraoral route.

## Introduction

The human skeleton mostly has deposition of calcium salts, mainly calcium phosphate. If this type of deposition occurs in soft tissue in an un-coordinated way, then it is known as heterotrophic calcification and categorized as metasta­tic, dystrophic, or idiopathic. Minerals depositing into normal tissue because of higher than normal serum levels of phosphate (chronic renal failure) or calcium (hyperparathyroidism) is known as metastatic calcification. The calcium deposition in normal tissue despite normal serum phosphate and calcium levels is known as idiopathic calcification. The pathologic calcification that occurs in dead and degenerative tissue or scarred tissue despite normal levels of serum phosphate and calcium is known as dystrophic calcification [1]. Here we present a case of unusual location of dystrophic calcification in the oral cavity.

## Case presentation

An 18-year-old patient reported with dysphagia and sensation of foreign body in the throat region on the left side. Difficulty in swallowing solid food was present, while there was no difficulty for swallowing semi-solid and liquid diet. The patient gave a previous history of infection in the left mandibular angle region which was treated with incision and drainage eight years back. The patient does not recollect any history of trauma in that region. The patient was healthy, and her blood investigation values were within normal limits.

**Figure 1:**
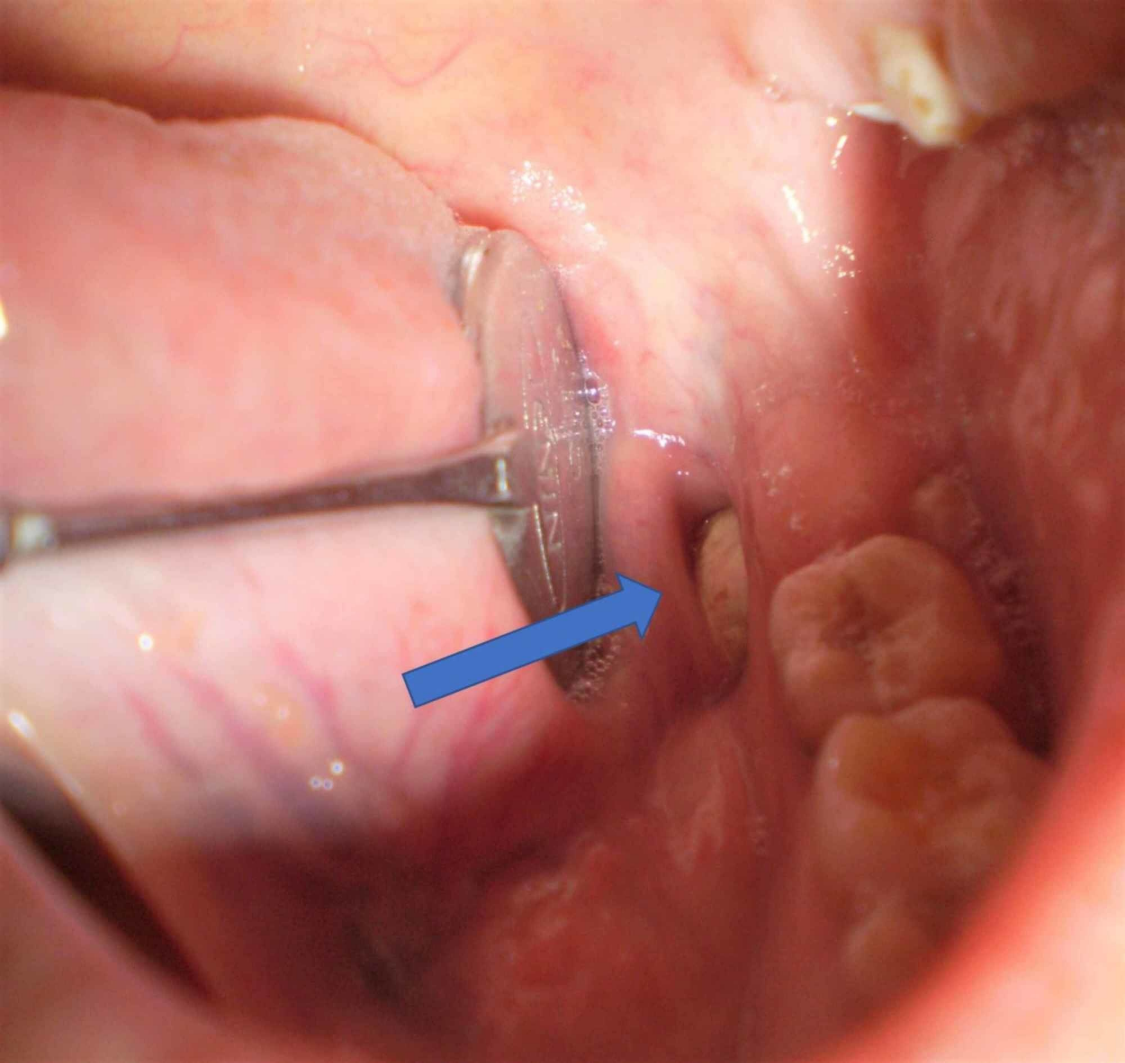
Intra-oral view Picture showing the calcified mass in the floor of the mouth.

Extra-oral examination revealed an old linear scar in the left mandibular angle region. Intra-oral examination revealed a yellowish-white mass in the lingual sulcus anterior to left anterior faucial pillar and distal to left mandibular third molar region (Figure 1).

There was no evidence of inflammation of surrounding tissues. The mass was firm on palpation and was not associated with pus discharge. The patient did not have problems in salivation or symptoms of ductal blockage.

**Figure 2:**
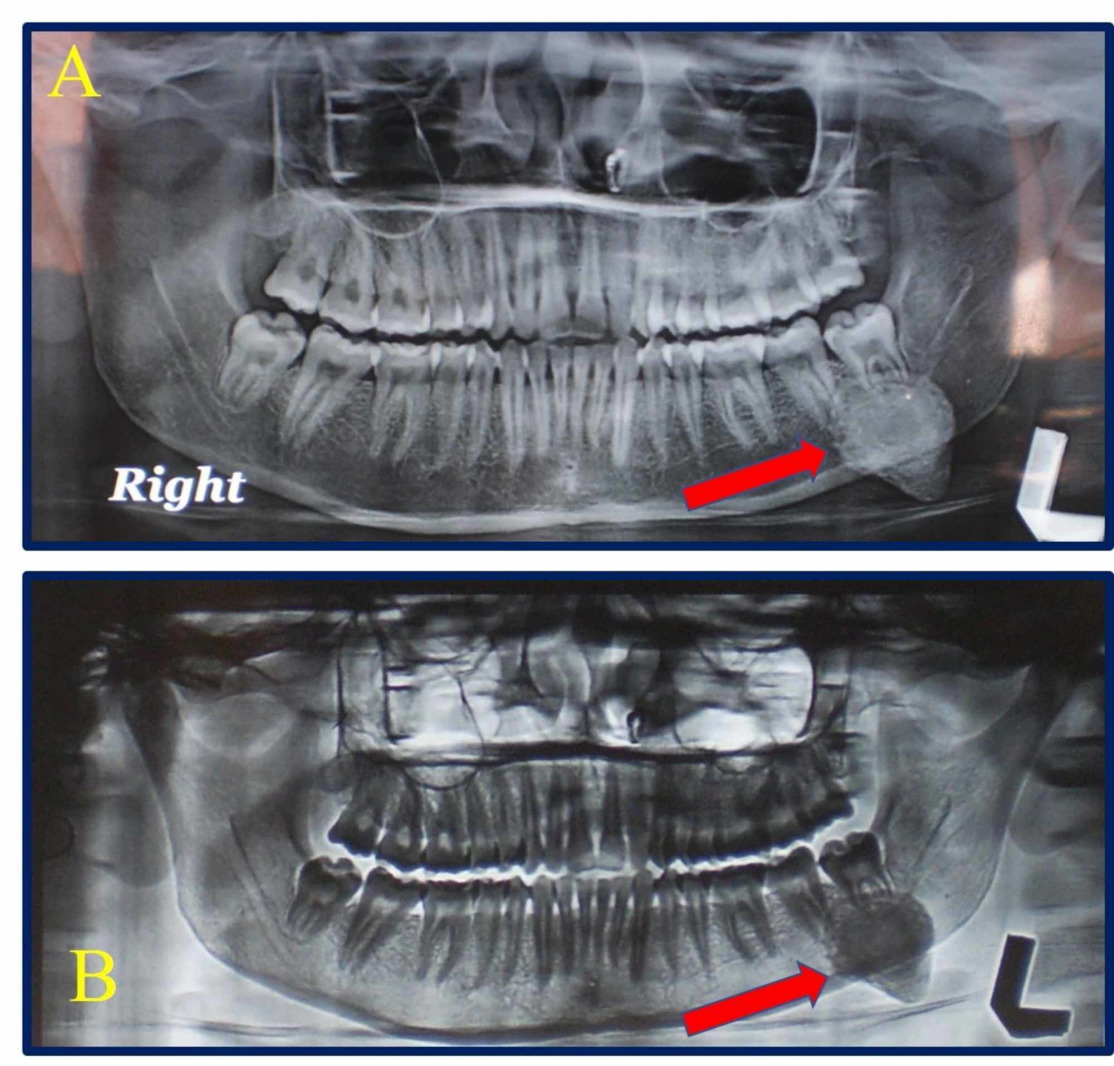
Panoramic X-ray (OPG) (A) Shows the OPG with the pear-shaped calcification. (B) Shows the negative (Inversion) image of OPG with the pear-shaped calcification.

**Figure 3:**
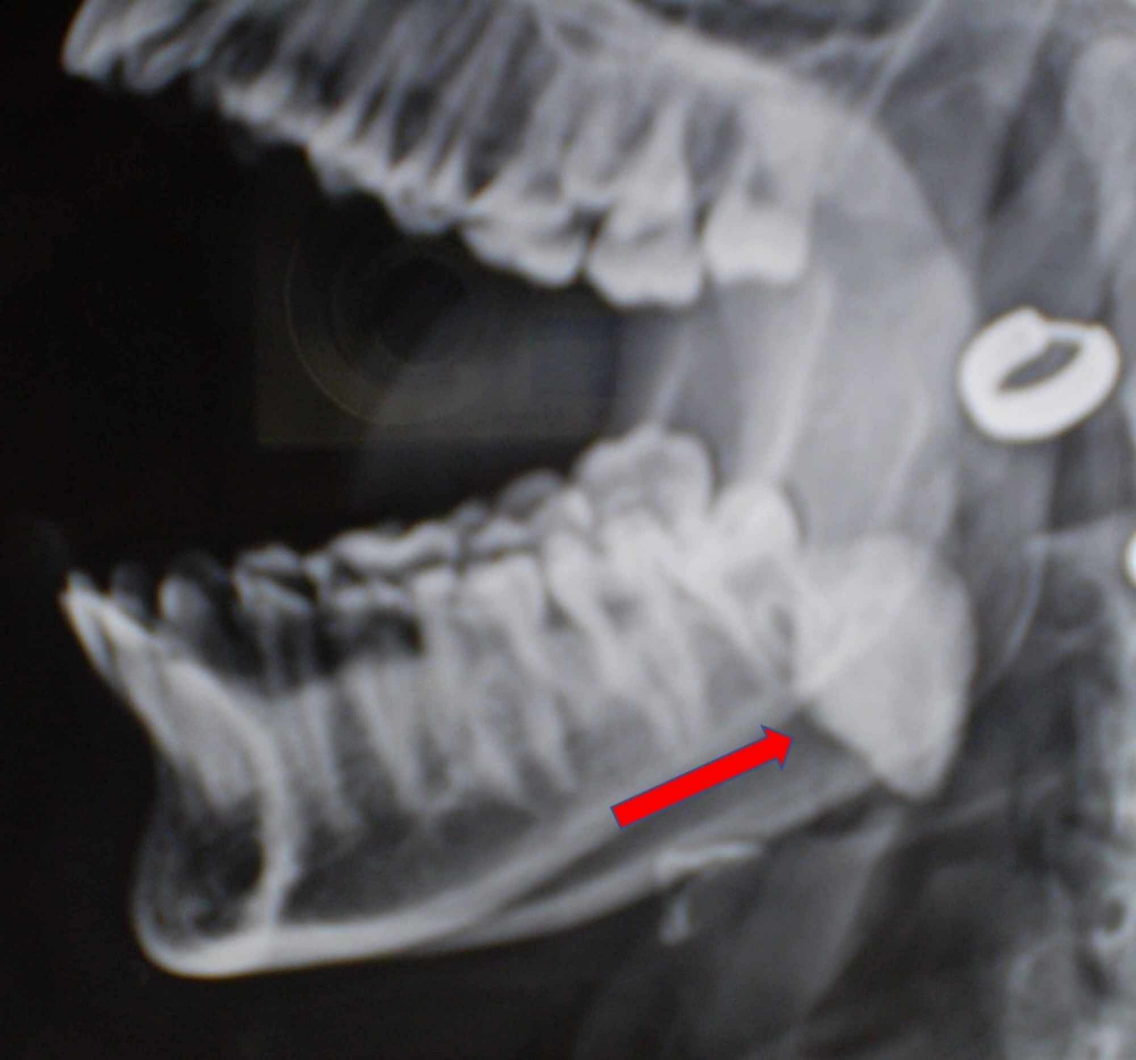
Lateral jaw radiograph

Radiographic examination (OPG) (Figure 2A, 2B) and lateral jaw (Figure 3) revealed a pear-shaped radio-opaque mass overlying the roots of 38 and distal root of 37 and extending below the lower border of mandible. 37 and 38 teeth were found to be vital and no tenderness to percussion was present.

The removal of this calcified mass was carried out intraorally under local anesthesia (2% lignocaine with epinephrine 1: 80000). An incision was given on the floor of the mouth and by extending the opening of the lesion, total removal of the mass was carried out. Hemostasis was achieved, and closure was carried out using 3-0 vicryl.

## Discussion

The calcium salts deposition in the tissue other than enamel or osteoid is called heterotrophic or pathological calciﬁcation [2]. When deposition occurs locally in dying tissues, it is known as dystrophic calciﬁcation or calcinosis [3].

Dystrophic calciﬁcation is mostly seen in subcutaneous tissues secondary to infection or trauma and is described in systemic lupus erythematosus, scleroderma or dermatomyositis. Injured tissue of any kind may be predisposed to dystrophic calciﬁcation [2]. In our case, the patient had a previous history of infection near the mandibular angle region and incomplete treatment could have resulted in the calcification.

Tonsillolith was one of the differential diagnoses. Tonsilloliths are calcium salt deposits arising from material retained in the tonsillar crypts and occur most frequently in young adults affected by recurrent upper airway infections [4]. It may also originate from a mass of retained material and bacterial colonies [5]. There was no evidence of frequent upper respiratory tract infection (URTI) and radiographically the mass was located more towards the mandibular angle region.

The differential diagnosis considered was calcifications in the lymph nodes, carotid artery, metastatic, stylohyoid ligament and, salivary gland.

Carotid artery calcification is mostly located in the soft tissue beneath the mandibular angle and between the cervical spine image and the hyoid bone [6].

The pathological condition caused by the deposition of calcified products in normal tissues because of hypercalcemia with or without hyperphosphatemia is called metastatic calcification [7].

The lymph nodes calcification commonly occurs beneath or near the angle of the mandible in the submandibular region. On radiographic examination, these calcifications mostly appear as well-defined, irregularly shaped opacities and can be described as “cauliflower” like. One prospective method to differentiate between lymph node calcification and sialolith is that submandibular sialolith is most frequently solitary whereas calcified lymph nodes are often multiple [8].

Standard occlusal projections and panoramic radiographs of sialoliths reveal that they are most common in the submandibular glands (83% to 94%), followed by the parotid gland (4% to 10%) and finally sublingual gland (1% to 7%). Patients with sialolithiasis may be asymptomatic, or they may develop obstructive sialadenitis characterized by pain and periodic swelling, especially around mealtime when salivary flow is stimulated. But these symptoms were not seen in our case.

The panoramic image of the ossified stylohyoid ligament extends from the mastoid process crossing the posteroinferior aspect of the ramus and towards the hyoid bone [9]. The stylohyoid ligament calcification mostly occurs bilaterally and proceeds downward from the base of the skull. In most cases, the individuals are asymptomatic, and treatment is not advised. Eagle’s syndrome is associated with calcified stylohyoid ligament. Vague deep throat pain on swallowing, opening the mouth, turning the head and otalgia (earache) are the symptoms [10].

## Conclusions

The deposition of calcium salt in degenerated tissues in the absence of a systemic mineral imbalance is called dystrophic calcification. It is mostly associated with trauma, inflammation or infection, and rarely appears in the head and neck area.

Different types of calcifications may occur in the head and neck region, and they need to be distinguished from dystrophic calcification by thorough history taking, clinical examination together with blood testing and radiographic examinations.
